# Studying microbial functionality within the gut ecosystem by systems biology

**DOI:** 10.1186/s12263-018-0594-6

**Published:** 2018-03-06

**Authors:** Bastian Hornung, Vitor A. P. Martins dos Santos, Hauke Smidt, Peter J. Schaap

**Affiliations:** 1 Laboratory of Systems and Synthetic Biology, Wageningen University and Research, Stippeneng 4, 6708 WE Wageningen, the Netherlands; 2 Laboratory of Microbiology, Wageningen University and Research, Stippeneng 4, 6708 WE Wageningen, the Netherlands

**Keywords:** Microbiome, Systems biology, Modelling, NGS, Metagenome, Metatranscriptome, Genome scale metabolic model, Gut, Community interactions, Microbial ecology

## Abstract

Humans are not autonomous entities. We are all living in a complex environment, interacting not only with our peers, but as true holobionts; we are also very much in interaction with our coexisting microbial ecosystems living on and especially within us, in the intestine. Intestinal microorganisms, often collectively referred to as intestinal microbiota, contribute significantly to our daily energy uptake by breaking down complex carbohydrates into simple sugars, which are fermented to short-chain fatty acids and subsequently absorbed by human cells. They also have an impact on our immune system, by suppressing or enhancing the growth of malevolent and beneficial microbes. Our lifestyle can have a large influence on this ecosystem. What and how much we consume can tip the ecological balance in the intestine. A “western diet” containing mainly processed food will have a different effect on our health than a balanced diet fortified with pre- and probiotics.

In recent years, new technologies have emerged, which made a more detailed understanding of microbial communities and ecosystems feasible. This includes progress in the sequencing of PCR-amplified phylogenetic marker genes as well as the collective microbial metagenome and metatranscriptome, allowing us to determine with an increasing level of detail, which microbial species are in the microbiota, understand what these microorganisms do and how they respond to changes in lifestyle and diet. These new technologies also include the use of synthetic and in vitro systems, which allow us to study the impact of substrates and addition of specific microbes to microbial communities at a high level of detail, and enable us to gather quantitative data for modelling purposes.

Here, we will review the current state of microbiome research, summarizing the computational methodologies in this area and highlighting possible outcomes for personalized nutrition and medicine.

**Electronic supplementary material:**

The online version of this article (10.1186/s12263-018-0594-6) contains supplementary material, which is available to authorized users.

## Background

The gut is an essential part of the human body. It has so much influence on our well-being that it even has been dubbed a “second brain” by the media [1, 2], and in recent years this “superorgan” inhabited by trillions of microorganisms has triggered a large amount of scientific interest.

**Fig. 1:**
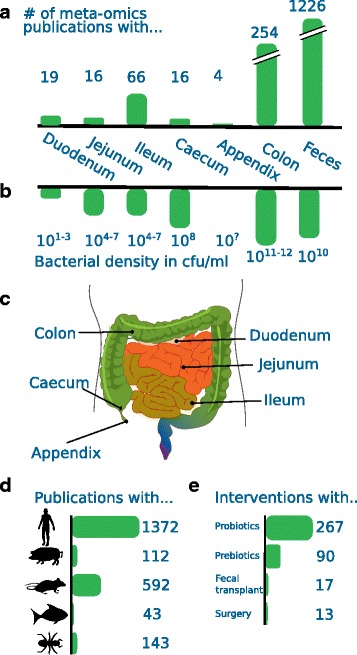
The gut in the focus of meta-omics science. An overview of **a** main sampling sites and **b** microbial complexity is given, together with **c** an overview over the physiology. **d** The number of the studied hosts and **e** methods to improve gut health are indicated. All data was retrieved via PubMed searches for the corresponding terms. For the exact search terms, please see Additional file 1

The microbial communities residing in the different parts of the gut are among the main contributors to its functioning and therefore also directly influence health. The recent availability of high-throughput methods (metagenomics and other omics) have improved our insights into these ecosystems dramatically. Figure 1 summarizes the current state of meta-omics (all nucleotide sequencing approaches, as well as metaproteomics and meta-metabolomics) research with an intestinal focus (for details regarding the literature search methodology, see Additional file 1). Not surprisingly, the largest body of research has been focused on humans (Fig. 1d), but other (model) organisms including pigs, rodents (mice, rats) and fishes (mainly zebrafish) have also been investigated. Non-model organisms are also under investigation, but for different purposes such as the potential biotechnological applicability of lignin degradation by termite gut microbial species [3].

Over the trajectory of the human gut, the microbiome has a varying degree of complexity [4, 5] (Fig. 1b). In general, microbial density increases from the duodenum until it reaches its maximum in the colon and faeces. At the same time, these two parts are also the most studied parts (Fig. 1a). While the high complexity of the community at these specific sites makes them interesting research sites, other parts of the (healthy) human gut remain grossly under-sampled, which is mainly due to inaccessibility. Along the trajectory of the human gut, the focus of microbial metabolic activities changes profoundly, with the small intestine having a higher capacity to degrade simpler carbohydrates [6], whereas in the colon mostly complex carbohydrates are degraded [7].

Most human omics studies are observational, aimed at studying microbial diversity and function as well as host-microbe interactions; however, a number of studies directly aim at improving gut health (and in proxy, individual health, Fig. 1e). These interventional studies can be broadly classified into two categories: pre-clinical and clinical interventions. Pre-clinical interventions focus mostly on improving gut health via changes in nutrition. In this field, the concept of probiotics (administering of beneficial bacteria [8]) is probably the most widely known, also in the eye of the general public, due to a wide array of commercially available products. Most interventional studies have focused on these probiotics, with a smaller part investigating the benefits of prebiotics (substrates enhancing the growth of beneficial bacteria in the gut; for a review, see [7]). Clinical interventions in response to conditions associated with a chronic disruption of intestinal homeostasis such as ulcerative colitis, and IBS with for example faecal transplants and bariatric surgery, have only been reported in a few publications [9, 10].

With all these studies, many important factors have been discovered regarding the ecology of the human microbiome.

### The human microbiota: symbiosis, competition and other relationships

Our microbiota is an important part of our personal ecosystem, which is assumed to be composed of more than a trillion microbial cells [11], approximately equalling the amount of human cells in our body [12]. Whereas the microbial ecosystems associated with some niches of the human body like for example the vagina [13] have a low complexity with only a few different inhabitants, most body sites contain hundreds of different microbes [11]. Like in macro-ecology, they perform different roles and thus can have different relationships with each other and with the host. In the microbiota, a broad range of different interactions exist, ranging from mutualistic and commensal to predatory relationships, and competition for the same niche exists. The nature of these relationships has an impact on the habitat itself, and imbalances with respect to the abundance and function of specific members can lead to an imbalance of the whole ecosystem. Many bacteria like for example *Akkermansia muciniphila* [14] have a good symbiotic relationship with their host. They degrade the carbohydrates supplied by the host, and other bacteria benefit from the breakdown products of this degradation process. This leads to the production of host beneficial compounds like short-chain fatty acids (SCFA; mainly acetate, propionate, butyrate) [15], which can be for example used by human colonocytes as energy source [16] or directly be incorporated into the human metabolism as additional carbon sources [17]. In other cases, this symbiosis applies to nutrition-derived carbohydrates that are not (fully) digested by host-derived enzymes in the small intestine such as resistant starch and other complex carbohydrates [7]. These might only be broken down by specific combinations of microorganisms for further catabolization. This can be exemplified by consortia of Bifidobacteria [18], which lead to the liberation of otherwise inaccessible substrates from for example indigestible plant biomass like cellulose components. In both scenarios, the liberated substrates can be further metabolized by other bacteria (e.g. [19]) to host beneficial compounds. Parasitic relationships also exist, like for example between *Actinomyces odontolyticus* and TM7 [20], where the parasitizing TM7 might eventually kill its microbial host. There are also predatory relationships, e.g. bacteria of the genus *Bdellovibrio* prey on other bacteria as source of energy and therefore help to regulate the diversity and balances of bacterial populations [21, 22]. Imbalances in the ecosystem might lead to bacterial overgrowth, which makes the ecosystem in general less resilient to perturbations [23]. Blooms of bacteria, e.g. *Clostridium difficile*, which infects more than half a million individuals per year and leads to 29,000 deaths in the USA alone [24], will have a directly noticeable impact. The produced toxins in such an outbreak will not only affect the microbiota [25] but will also lead to a direct disease state of the host [26]. Therefore, understanding of internal and external factors that affect composition and functioning of this ecosystem, such as for example nutrition intake, antibiotic intake, symbiotic or predatory relationships, are essential for being able to characterize and predict the state and functioning of this ecosystem. All of these challenge the intrinsic emergent community properties such as resilience, stability and its efficiency to provide nutrients for the host.

#### Metabolic syndrome and the microbiome

The metabolic syndrome is a complex disorder with high associated cost and is mainly characterized by four sub-pathologies: Obesity, elevated blood sugar/insulin resistance/diabetes type II, elevated blood pressure and dyslipidemia [27, 28]. Although genetics [29] and lifestyle [30] play major roles, the microbiome also contributes to all of these main sub-pathologies.

Obesity might provide the most direct link. It has been shown that gut microbiota composition in obese and lean individuals is significantly different [31]. The microbiome is an important factor in carbohydrate degradation and uptake. Microbial metabolism on average contributes to up to 10% of the daily calorie intake [32], and potentially in obese subjects, this contribution could be increased [33]. This is mainly due to the degradation of carbohydrates, which due to the lack of necessary catabolic enzymes, are not directly accessible for the human host. These carbohydrates are converted by the microbiota into SCFA, thereby directly contributing to the energy intake of the host [34]. Since not all microorganisms are capable of such conversions, species diversity and abundance will directly influence the types of carbohydrates that can be converted into SCFA and therefore how much of the non-digestible carbohydrates will be utilized by the host-microbe holobiont. While some bacteria are specialized in carbohydrate breakdown, like for example *Bacteroides thetaiotaomicron* [35], others mainly rely on their peers to scavenge nutrients [36]. A microbial community consisting mainly of carbohydrate degraders will therefore be more beneficial for the host providing valuable nutrients. It is tempting to speculate that in case of obesity this beneficial trait has turned disadvantageous and might contribute to an increased risk towards metabolic syndrome-associated pathologies.

Such differences in microbial composition have also been causally linked to obesity. It has been shown that transplantation of an “obese microbiome” into germ-free animals causes an increase in body fat as compared to control animals inoculated with a “lean microbiome” [33, 37, 38], indicating that the increased capacity to harvest energy is transferred with the microbiome.

The involvement of the gut microbiome in the second most prevalent pathology, elevated blood sugar/insulin resistance leading to diabetes type II, can be explained via an indirect route, starting from inflammation. Even without an obvious disease phenotype, low-grade inflammation might be present [39], caused by yet unidentified bacteria. This inflammation is hypothesized to be one of the causes of the metabolic syndrome [39, 40] and to be an early stage of Inflammatory Bowel Disease, including Ulcerative Colitis and Crohn’s Disease [41]. An invasion of bacteria into the intestinal tissue causes the presence of endotoxins (LPS, flagellin) in the blood stream, leading to chronic inflammation in the intestinal tissue. It has been suggested that as a physiological response to inflammation the blood glucose level is increased to serve as additional energy source for the various immune cells [42]. Since the inflammation is chronic, so will be the elevated glucose levels. In the long term this might lead to insulin resistance and type II diabetes [43].

The connection between the composition of the human gut microbiota and the third and fourth pathology, elevated blood pressure and dyslipidemia, is less well characterized [44]. It has been demonstrated with cross-over experiments that gut microbiota from rats with elevated blood pressure will transfer this physiological trait to receiving rats [45]. It has also been shown that inflammatory processes [46] and effects on the nervous system [47] will affect blood pressure, but a full understanding of these relationships is still missing. For dyslipidemia, the relationship is also rather unclear, due to its strong association with obesity [48]. The clearest mode of action until now are effects of the microbiota on bile acid metabolism, which is critical for the absorption of lipids [49], but the observed associations are currently not linked to known mechanisms [50, 51].

### Top down: how to investigate the microbiome

In contrast to macro-ecology, in microbial ecology, it is possible to capture nearly the whole biodiversity of a habitat by sequencing its associated total DNA and/or specific phylogenetic marker genes. Different omics techniques can give the researcher information about species diversity and abundance, about their metabolic capabilities and associated symbiosis or pathogenicity factors. Technically, there are different ways of obtaining this information but the ultimate goal of omics approaches is to answer the following set of questions: Who is there, what can they do and what are they actually doing?

While in macro-ecology, specimen can normally be collected and studied in captivity; this is usually not the case for microbial ecosystems. It is assumed that we can only cultivate less than 1% of the bacterial diversity [52]. The rest, the so called “dark matter” cannot be readily captured by cultivation [53], although much progress has been made in recent years with high-throughput culturing, the so called “culturomics” [54]. While bacteria make up most of the diversity of the human microbiota, archaea are also present in humans [55], as well as a high diversity of phages [56]. Fungi and protozoa also exist in this ecosystem, but are less well studied [57]. Why the majority of this biodiversity cannot be cultured is not clear, but different hypotheses exist. One of these hypotheses is that these organisms cannot survive on their own because of community dependencies. They are for instance microorganisms that live in a strict syntrophic relationship and are sharing nutrients and metabolites [58]. Syntrophic relationships might be due to excretion and uptake of common metabolites, but also more intricate cross-feeding networks have been reported to exist [6, 59, 60]. Other types of non-metabolic interactions also exist but are less easily quantifiable. Biofilms, which occur frequently in human-associated microbiomes [61], are often not the product of a single species, but of a community [62]. They are not controlled by direct metabolic dependencies but by other mechanisms like quorum sensing [63].

### Omics approaches towards understanding of the who and what of microbial communities

To answer the “who”, the “what can they do”, the “what are they actually doing” and “how do they respond to a diet or otherwise environmental change”, different approaches can be used. To answer the “who”, low-cost amplicon sequencing of 16S ribosomal RNA (16S rRNA) encoding genes can be utilized. The 16S rRNA gene is present in all prokaryotes and slowly mutating due to structural and catalytic constraints. Some of the secondary structure elements, called regions V for variable 1 to 9, are less constrained and therefore over time accumulate mutations more rapidly than other more conserved regions. Together, sequence variation within conserved and variable regions can be transformed into an evolutionary distance, allowing interference of the phylogeny of all members within a microbial community. As knowing the community composition in most studies is a prerequisite, next generation sequencing (NGS) of PCR amplicons targeting a selection of these variable regions is the most widely used approach. Despite the fact that no genomes are sequenced, this is often falsely referred to as “metagenomics”. This should be avoided and proper terminology should be used [64]. Nevertheless, making use of the currently available information from genomes and metagenomes, species identification in part also allows for predictions of functional capabilities [65, 66], albeit with inherent limitations with respect to their accuracy especially for understudied environments that are less well represented in currently available (meta)genome databases [67]. To more comprehensively answer the question “what can they do”, metagenomics can be used. Metagenomics significantly increases both the amount and the complexity of the data. Besides the “who”, and the “what can they do”, community responses to diets or otherwise environmental changes can be studied by metatranscriptomics to answer the question “what are they doing”. Sequencing the full transcriptome of the community provides by proxy insights in which pathways/processes are actually active. The logical progression of technology also leads to metaproteomics, which due to lack of precisely matching reference genomes [68] is still not very widely used and despite interesting results [69, 70] still remains to represent a niche discipline [71]. Meta-metabolomics (also called metabonomics [64], although this term has been used for a different purpose [72]) is currently an even less used technique.

**Fig. 2:**
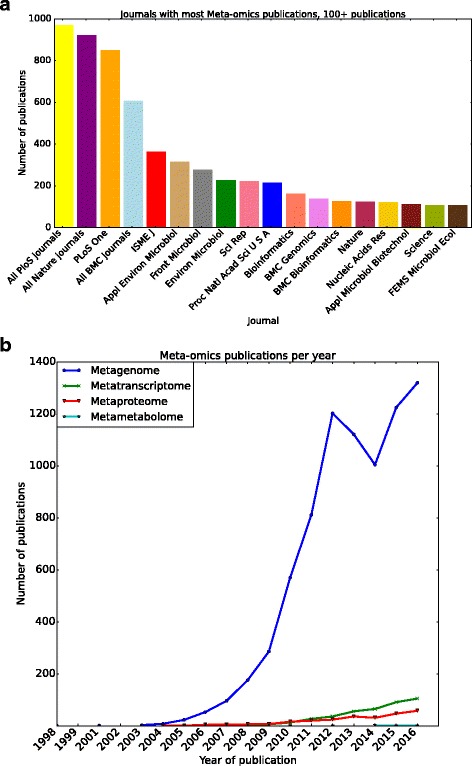
**a** Journals with the most gut-related meta-omics publications. **b** Overview of gut-related omics publications per year. 16S rRNA gene sequencing and metagenomics are combined, since these cannot be easily distinguished via title/abstract searches due to the erroneous labelling of amplicon sequencing approaches as metagenomics by many researchers. All data was retrieved via PubMed searches for the corresponding terms. For the exact search terms, please see Additional file 1

A large body of research applying abovementioned omics approaches is published in well-known journals. Figure 2a provides data up and until 2016. PubMed lists after the initial publications starting in the early 2000s an increasing amount of publications per year, reaching to more than a 1000 per year at the moment (Fig. 2b). The focus of most of these publications is on DNA-based approaches, including 16S rRNA gene sequencing and true metagenomics. This trend is followed distantly by metatranscriptomics, metaproteomics and meta-metabolomics. Since by far the majority of these publications are within the scope of some form of high-throughput nucleotide sequencing (16S rRNA gene, metagenomics, metatranscriptomics), in the following paragraphs, we will focus on these omics approaches.

### Differences within the omics technologies

The methods used for amplicon sequencing, metagenomics and metatranscriptomics are summarized under the term NGS technologies (also called second generation technologies; for a review see [73]), including highly automated technologies represented by Illumina sequencing machines like HiSeq or MiSeq, the Roche 454, Ion Torrent and SOLiD technologies. These technologies are a follow-up of Sanger sequencing, which still has the highest level of accuracy but has a rather low throughput due to limited parallelisation possibilities. NGS technologies allow millions of fragments to be sequenced in a single run. The DNA is randomly sheared, and all resulting fragments are sequenced with fluorescent nucleotides, which emit at incorporation in the new formed DNA strand certain light wavelengths. These can automatically be recorded by current systems and allow high-throughput sequencing information by generating millions of short reads. One lane on a typical Illumina HiSeq machine can generate up to 360 million reads, currently with lengths up to 350 bases. The limitation in this approach is mainly the used DNA polymerase for the extension of the newly formed DNA fragments, which tends to lose precision with increasing read length, making longer reads more error prone. Especially in metagenomics obtaining longer read lengths is important. Besides providing more information per single read, which is in general desirable in many cases, specifically for metagenomics it will (i) lead to a higher chance of uniquely assigning reads to a single microbial taxon leading to a better resolution in strain and species separation, (ii) make it easier to capture gene functionality and (iii) allow for a higher confidence during the assembly of the data, especially in those cases when the community harbours phylogenetically close species.

The new sequencing technologies (third generation sequencing) from Pacific Biosciences (PacBio) and Oxford Nanopore are ameliorating this problem. Both technologies can produce very long reads, up to 60,000 bases (PacBio) and more (Nanopore). PacBio circumvents the loss of precision of the polymerase by repeatedly sequencing the same DNA fragment [74]. Oxford Nanopore channels single-stranded DNA through a pore which carries an electric current, and measures the change in current as the DNA passes by, with each of the bases causing a different change. This technology does not lose precision with increased length, but generating longer fragments and stably channelling them is the limitation [75]. Current drawbacks of both technologies as compared to the second generation technologies are a higher error rate, requirement of a significantly larger amount of template DNA and higher sequencing costs. PacBio [76–81] and Oxford Nanopore [82] have already been used in microbiota sequencing and their use will most likely increase when the technologies further mature.

### Extraction of information from 16S rRNA amplicon sequencing data

The 16S rRNA molecule shows a high degree of structural and sequence conservation in all prokaryotic organisms. Being part of the ribosome, it is a crucial part of the translation machinery. Because the specific secondary structure and function constraints evolutionary drift, it is, albeit with some limitations [83], possible to work with “universal” or species-independent primers and therefore amplicon sequence analysis remains the standard approach to investigate microbial diversity. If two or multiple complete rRNA gene sequences have more than 97% identity, they belong to the same species. The 97% identity threshold is due to historical reasons because this value was found to be in agreement with DNA-DNA hybridization results, but otherwise no coherent species definition exists [84, 85]. In order to make clear that the actual species/genotype is often not known and might actually differ, 97% identity clusters of rRNA sequences are also referred to as “operational taxonomic units” (OTU).

The 16S rRNA gene is approximately 1500 nucleotides in size and for the highest confidence the complete sequence is required. Due to the read length limitations of second generation technologies researchers have therefore investigated, which sequence range of the rRNA showed the highest degree of variability and will therefore result in the best resolution [84, 86]. Using second generation sequencing techniques, these regions (variable regions V1-V9) are therefore preferentially sequenced (for a review see [87]). Here, region-primer combinations need to be carefully matched as these choices can have a high impact on the results [88].

In eukaryotes, like for example fungi, the situation is more complicated. Sequencing 18S rRNA genes does not provide the required resolution, and often internal transcribed spacers (ITS) are sequenced instead [89].

After the amplicon sequencing data has been generated, the next step is to derive corresponding information regarding community composition. In general, since sequencing of single phylogenetic marker genes (fragments) requires less throughput than whole genomes, also the costs per sample are considerably lower, providing the necessary statistical power for a more detailed analysis [90].

Using second generation sequencing techniques, there are multiple considerations involved, e.g. how similar the sequences are expected to be in the variable regions of choice, which reference database to use (SILVA [91], RDP [92] or Greengenes [93]), the significance of base-calling error rates intrinsic to high-throughput sequences data [94] and how erroneous sequences can be detected. Due to these challenges, sophisticated pipelines for taxonomic assignment have been developed, like for example Qiime [95], Mothur [96], Phyloseq [97], MICCA [98] and NG-Tax [99], the latter of which has been developed in our laboratories and provides computationally efficient and accurate taxonomic assignments and quantification of OTUs per sample with improved robustness against choice of region and other technical biases associated with 16S rRNA gene amplicon sequencing studies.

A range of different methods coming from macro-ecology is used to investigate a habitat’s diversity. The species richness or mean species diversity of a sample is often referred to as alpha-diversity and the amount of variation in species composition among the samples (beta-diversity) can also be investigated. A range of different alpha-diversity measures is being used, including those that account for species richness (defined as the absolute count of individual populations per habitat), phylogenetically weighted richness (Faith’s Phylogenetic Diversity [100]), and species diversity, including Shannon index [101] and Simpson index [102] (for a review, see [103]). Diversity indices also try to incorporate the evenness of the species distribution [104] because different conclusions need to be drawn if an ecosystem is dominated by a single species with a plethora of other rare species, or if the distribution is rather even. Another important aspect is under-sampling. To estimate if the true richness of species has been captured, different methods like rarefaction analysis, Chao1 [105] or ACE [106] estimators can be used (for a review, see [107]).

Analyses of beta-diversity make use of a number of different measures of pairwise community similarity, including for example Jaccard index [108], Bray Curtis dissimilarity [109] and UniFrac distance [110], the latter of which is phylogenetically weighted.

In most cases, a first look at the data is done with unconstrained multivariate statistical approaches such as Principle Component and Principle Coordinate Analysis (PC(o)A). These two methods try to fit highly dimensional data (e.g. a high amount of samples and different species in them) into a plot with two (or three) dimensions, trying to display as much of the variation in the data as possible. Factors that are potentially related to the observed variation, including for example environmental conditions, time points or the objective of the research, can be projected a posteriori, and their significance can be tested post hoc.

Several of these statistical tools are standardly embedded in sequence analysis pipelines like Mothur [96], Qiime [95] or Phyloseq [97] and allow to capture measures of alpha- and beta-diversity. Choices can be made between default analysis routines and more customized procedures where users can adjust specific settings.

With these methods, it has been found that for example the alpha-diversity in the microbiota of obese subjects is significantly reduced in contrast to the alpha-diversity in lean subjects [111]. Other successful studies in this field have already revealed that gut microbiota is transmitted vertically and that obese mice have a considerably less diverse microbiota than their lean counterparts [112]. Furthermore, it has been shown that the gut microbiota changes during human development starting at birth and is different depending on geographic location [113], during long-term dietary interventions [114] or when consuming specific diets even during a single day [115].

### Extraction of functional information from metagenome data

**Fig. 3:**
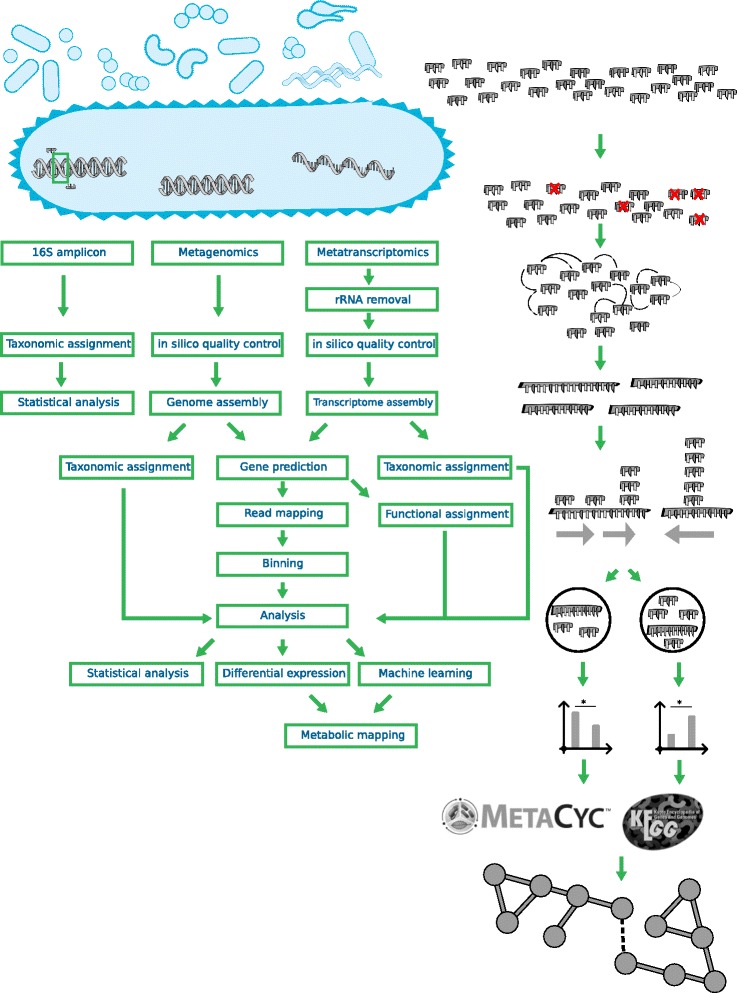
Overview of the different steps in the meta-omics analysis workflow. The different workflows are depicted, from left to right for 16s amplicon data, metagenomics data and metatranscriptomic data. The main steps for 16s amplicon data is the definition of OTUs together with taxonomic assignment, followed by statistical analysis. For metagenome data, first steps involve quality control steps, followed by a metagenome assembly. The workflow splits afterwards into two directions, one being the taxonomic assignment, the other one the definition of metagenomic bins and the functional annotation. Genes can be predicted from the genome assembly, which can be functionally profiled. With the coverage information of the genes, it is also possible to define genome bins. After this step is done, the same statistics as for 16s amplicon data can be performed, as well as differential expression/abundance analysis together with pattern detection through machine learning, and finally analysis of the metabolism. The workflow for metatranscriptomic data is in general the same, except that rRNA, which does not provide any information in this setting, needs to be removed before most of the steps, and that no binning is possible with transcriptome data

In principle, full genomic information can be captured with metagenomics. Seminal projects in this area like MetaHit [11] and the human microbiome project [116] made great efforts to sequence the metagenomes of diverse cohorts with many subjects to investigate the full functional capacity of the different microbiomes. The amount of data required makes deeper sequencing necessary, which complicates the workflow to extract information from metagenomics data (Fig. 3).

High-throughput sequencing data is noisy, and quality control is a critical first step (review see [117]). One crucial step for which settings have not yet been universally agreed upon is the quality trimming [118], and no consensus advice can be given.

For simple read mapping there are a number of strategies that can be applied. BLAST [119] or Diamond [120] can be used to match reads directly to KEGG, to quantify the functions based on the number of matching reads (e.g. applied in [38]). A higher resolution is obtained when reads are mapped to a set of reference genomes [111, 121], which also allows for a taxonomic classification of observed functions [122]. If the phylogenetic distance between the reference set and the sample is small this has the advantage of speeding up the analysis. Furthermore, associated functional annotations can be directly utilized, making a separate annotation step unnecessary. A major drawback for this type of workflow is that only known species can be analysed, whereas new strains with novel functions, horizontal gene transfer and other evolutionary events will not be captured, and micro-diversity will be lost.

An alternative approach therefore is to assemble reads into larger contigs and extract genomes directly from metagenome data [123] (Fig. 3). Today obtaining a high quality single genome can still be a challenge [124], and with a community genome assembly approach these challenges can multiply. Examples are chimeric assemblies between genomes due to presence of multiple strains of the same species (although miss-assemblies should not occur very often [125]), and a low coverage of low abundant species. At this point, it is also important to consider the mapping rate after the assembly. While we expect for a single organism that after the genome assembly most of the reads will map to the assembly, this can deviate for metagenomics. This is mainly due to the species richness and species evenness of the community under investigation. A complex species-rich sample of high evenness (i.e. similar abundance of many community members) will require more data to assemble the top-ranking species than a sample where a few high-ranking species have much higher abundances. Therefore species richness and species evenness need to be taken into account to evaluate if the mapping rate is appropriate for further analysis.

Some of these challenges have been tackled with specific metagenome assemblers like MetaVelvet [126], which take different properties of the sequencing data into account like for example the different abundances of the potentially present species. Currently, a community-derived assembly will also not lead to closed genomes. The next challenge is therefore to determine which of the assembled contigs/scaffolds belong to a single species. This process has been termed binning, and several tools such as MaxBin [127] or MetaCluster [128] have been developed to determine the amount of bins required and to assign contigs to bins. To do so, these tools take different types of information into account, such as k-mers frequency in the data or contig read coverage. The quality control of this step is critical, since this process is also error prone, especially when phylogenetically close organisms of similar abundance occur in a community.

The most widely used method to test for correctness of binning is based on single copy marker genes, like in for example CheckM [129]. Based on the presence of these necessary genes, both the coverage of a genome in a bin as well as the amount of contamination from other genomes can be determined. A problem with this approach is that it is limited to contigs/scaffolds containing these core functions.

Next the taxonomic origin of the various bins can be determined (Fig. 3). All programs and workflows which can perform this are reference based, but work with different mechanisms. One approach is to use BLAST [119] to compare all the metagenomic contigs against a database, like the NCBI NT database, or specialized databases like for example the human microbiome project [11]. The accuracy of the taxonomic assignments is proportional to the similarity score of the alignments. One of the first programs to deal with this problem is MEGAN [130], which also gives the user a graphical interface for direct analysis. The biggest drawbacks of this method are that (i) it can be computationally prohibitive to use a large database and (ii) that closely related species cannot be differentiated from each other. A computationally more efficient alignment free method for the taxonomy determination is to compare the k-mer profiles of the metagenomics contigs with k-mer profiles obtained from a reference database. This has been implemented in tools like Kraken [131] or PhyloPythia [132] (for a review of programs, see [133]),

To understand the underlying causes of a community change and potential effect, functional profiling needs to be performed (Fig. 3). This part of the analysis is for a metagenome mainly different to a single genome in regards to the quantity, but the basic processes are the same. First gene prediction needs to be performed with gene callers like example prodigal [134], which have special settings for this kind of data. A low-level profiling can be obtained with a COG analysis [135]. The COG ontology consists of limited number of broad categories, which allow the detection of extensive changes. When more data is available a higher resolution can be obtained. These can be for example (i) EC number prediction, which can be obtained via PRIAM [136] and can be linked to metabolic pathways using databases like KEGG or Metacyc [137], (ii) lists of carbohydrate active enzymes [138] can be obtained via dbCAN [139] and (iii) full domain profiles including GO terms [140] via for example InterproScan [141] or via second generation annotation tools [142]. With these so called full functional profiles, it is possible to reconstruct the metabolism of the bin [143–145], and bin-specific auxotrophies or special metabolic capabilities can be investigated. If someone wants to draw statistical conclusions for the difference in the metabolism by for example investigating for overrepresented functions (e.g. GO enrichment [146]), it should not be forgotten that, even for genomic information, replication is necessary [147]. If it is not possible to obtain all this data, due to lacking computational resources, also web services like IMG/M [148] or EBI metagenomics [149] can be used, which normally also have a user friendly interface, but only offer a limited depth of analysis.

### Extraction of functional information from metatranscriptome data

The transcriptome approach will allow the investigator to focus on functions that are actually expressed in a given sample. A highly abundant species may show a low expression of functions of interest and vice versa (e.g. [150]). In fact, since DNA is also highly stable, the metagenomics approach might also take non-viable cell populations into account, which could falsify the conclusions, but also separate measures, like removal of non-viable cells, can be taken to prevent this [151]. Thus, the metatranscriptome provides a more accurate account of actual functionality.

Most relevant steps, including QC, are the same as for single organism transcriptomics (for a review, see [117]; workflow, see Fig. 3). Not mentioned in [117], but necessary for metatranscriptome data is the in silico removal of spurious rRNA reads [152] as in vitro removal of rRNA prior to sequencing will most likely not remove all of it.

Like in metagenomics mRNA reads can either be mapped or de novo assembled. Mapping can be done if a set of reference genomes is available. If binning has been performed before, then the transcriptome should not be mapped to the different bins separately. If bins were separated before mapping, then the assignment of reads would be skewed if phylogenetically related bins are present (incorrect multiple assignment of reads). If no reference metagenome is available, it can be attempted to map the RNAseq data to related datasets. In this case again, the absolute mapping rate of the data needs to be cautiously taken into account, because an unsuitable reference (due to large phylogenetic distance or missing species) will exhibit low mapping rate and will prevent a full analysis of the data. Alternatively, a de novo transcriptome assembly can be performed. Specific metatranscriptome assemblers have been developed to deal with the complexity of such data (for a review, see [153]). Subsequent mapping of the same mRNA reads onto the de novo assembly allows for differential expression analysis, which can be performed with known tools like for example edgeR [154] or DESeq2 [155].

In many regards, metatranscriptome analysis can function as a substitute for a metagenomics analysis while adding an additional layer of information. For instance, metatranscriptome analysis has already revealed that activity of carbohydrate degrading enzymes can be underestimated if only genomic information is considered, or how the activity of the gut microbiome responds to different diets [156, 157]. In principle, similar conclusions could also be obtained from a combined metagenomics/metaproteomics approach [158] albeit at lower resolution.

A pure transcriptome assembly has the drawback that binning is not possible, since many of the binning approaches rely on the fact that in a metagenome all contigs from one species will exhibit similar coverage, which is not the case for a transcriptome. It will also not be possible to assemble very long contigs, because many intergenic regions will not be transcribed. Important changes at the ecosystem level can be assessed by analysing the expression levels of the microbiota in the community provided that species abundances are also taken into account; a 50% increase in abundance might appear as a 50% higher gene expression, but in this case does not reflect a transcriptional response on a per-microbe basis, but rather a compositional response at community level.

### From information to understanding

As exemplified above many computational tools and pipelines exist that are able to extract biological information from high-throughput data. Understanding the unique chemical and functional capabilities of the human microbiome and deciphering the biological roles of individual species is much more difficult. Linking microbial activities with gene expression and enzyme functionalities is just the first step. In early years of genomic research, “hairball” graphs had their appearance in many publications, showing connectivity within the available pile of data, rather than focusing on the biologically informative parts. With the increasing number of samples being analysed for example from patients, from replicates, from different conditions, different types of sequencing data combined with different types of computationally derived data such as EC number and domain predictions, which methods can be used to gain useful information?

The most obvious approach, especially with pure abundance data, is looking for correlations (also possible via regression [159]). It can be assumed that correlating species/OTUs have a symbiotic relationship with each other and/or with a third OTU, whereas anti-correlation can (but does not have to) indicate antagonistic behaviour. There are, however, several pitfalls. For example, OTUs, which are present only in very few samples, will be highly correlated due to the common absence in multiple samples. While this general conclusion can be true, it needs to be considered that absence in sequencing data does not have to mean absence of the organism. It can also indicate abundance below the detection threshold, or simply a failure in detecting the organism with the current pipelines.

The same methods described above for the analysis of 16S rRNA gene amplicon sequence data can also be utilized for metagenomics data. Multivariate visualization tools such as PCA can be used to see if specific sample groups, e.g. defined by specific interventions or states of health, cluster together, or if other factors are more prevalent in explaining the observed variation in the data. Nevertheless, for the in-depth analysis, more sophisticated methods should be used such as for example pattern recognition, which enables the researcher to find useful information in big data. This field is broadly classified into two approaches, i.e. supervised and unsupervised learning. In supervised learning, the researcher tries to classify unknown samples into categories for which already known samples exist. If, for example, samples from lean and obese subjects have been obtained, an algorithm can be trained to determine if samples of unknown origin were obtained from a lean or obese person. While supervised learning has been already used in microbiome research with great success, e.g. [160, 161] (for reviews of the methodologies, see [162, 163]), and is currently researched for the application in many different fields and termed “life changing” for the general public (e.g. deep learning [164]), this approach is often hampered by the fact that samples from different studies are not comparable due to different methodological approaches with respect to for example DNA extraction or sequencing method and depth.

Unsupervised learning, also called clustering, does not rely on prior information. Clustering algorithms, including hierarchical clustering, k-means and dbscan, try to find unknown patterns in the given data, e.g. different patterns of gene expression over multiple conditions. This approach has also been used for example to determine the enterotypes [165] but also suffers from a wide array of challenges. The choice of clustering algorithm is not trivial and depends on the structure of the data, which can often not be determined in an easy way [166]. Furthermore these algorithms often rely on user-defined parameters such as the amount of clusters to find. Determining the best parameter set is its own research field, given that more than 30 different algorithms for this purpose exist [167], and not all are applicable to all clustering algorithms [166]. If at the end, wrong parameters are chosen; it might lead to erroneous conclusions, like for example if not the optimal amount of clusters (in this case, enterotypes [168]) is selected. Otherwise, a cluster might be split into multiple, or multiple distinct clusters might be treated as one.

Having said that many of these algorithms have been implemented in different programs like ELKI [169] or WEKA [170] and can also be utilized by inexperienced users, although the final evaluation still often requires expert knowledge.

If useful patterns have been obtained after the machine learning, the last level is the biological understanding and interpretation. Simple approaches include just mapping extracted functional information such as EC numbers and KO numbers to pathway databases like KEGG [171]. More sophisticated solutions try to automatically extract the useful information from these networks, e.g. MetaModules [172]. If also other non-metabolic functions should be investigated, then a broader type of classification can be used. The most common analysis is the GO enrichment analysis, which aims to identify overrepresented functions in the dataset [146].

It also needs to be considered that the microbiome data does not have to stand on its own. If clinical or nutritional data is available, these can be used as well. Correlating such metadata with microbiome data has shown that factors like age or stool consistency are highly related to microbiome composition [173], as well as the hosts genetics [174]. Furthermore, it is also possible to revert this and use microbiome data together with clinical data to predict a persons’ glycemic response to food intake [175].

Since this type of data can be highly connected, visualization of this connectivity might be necessary for a better understanding. While some visualization forms are standard, for example depicting the distribution of species/OTUs per sample in a bar chart, and metabolic networks as networks, sometimes more sophisticated methods are necessary. For analysis purposes, the Krona library [176] can be a useful visualization tool to explore quantitative hierarchical relationships between taxonomical groups. In many cases, there are no standard recipes for the analysis workflow, and custom solutions have to be developed. For these cases it is necessary to consider what type of data should be shown, and with which method they are obtained. Several visualization methods are available [177, 178], but standard packages for many of these are not necessarily developed yet or easily accessible.

### Bottom up: mechanistic insights into the microbiome

The next step after collecting data and investigating the communities is building models and testing hypotheses. While with single species this is very well doable, microbial communities pose more challenges to the researcher. For a single culturable species, it will be possible to collect the necessary data. It is possible to reconstruct the full metabolism (according to current knowledge), manually curate it, and measure a vast array of metabolites. In contrast, all these factors pose challenges in a community like the intestinal microbiota.

### The sum is more than its parts

A community is more than an accumulation of multiple single organisms. The different microbes interact within a dynamic environment; they will behave differently, depending on who is in the surrounding, and what they are doing. Even for a single species, species abundance can lead to emergent properties for example via quorum sensing, which can alter the behaviour of individual cells and the entire population dramatically [179]. In biofilms, the formation itself is an emergent property, which would not be possible to observe if only single cells are considered. It also leads to the change in behaviour of the different cells, as some will get advantages in this environment (protection), whereas the cells on the surface are less protected, but also have more access to nutrients. Other forms of symbiotic relationships can also lead to emergent properties where for example some species in the community provide the means to overcome amino acid auxotrophies or vitamin deficiencies of others or of the host [180–182]. Another unrelated example from the oceanic microbiome is the detoxification the environment [183]. This case is commensalistic, since a big part of the microbial community benefits from the ability of one member to detoxify oxygen radicals, giving the other members a benefit, which lead in this case to genome streamlining by loss of genes related to oxidative stress. The authors even expanded their observation into the “Black Queen Hypothesis”, stating that this streamlining together with a dependency on helper organisms with leaky beneficial functions might be an universal concept. This is only possible to observe at the community level, and the investigation of a single species would not lead to such conclusions.

Numerous additional examples exist, also in the gut environment (for a more complete review, see [184]).

### How to predict the sum from its parts

How should the behaviour of such a community be predicted? The apparent approach is to model the metabolism of the whole community as a single entity or “supra-organism”, neglecting species boundaries [185]. While this can give an idea about the metabolic capabilities, it is an oversimplification and will miss critical steps like metabolite exchanges and interdependencies between organisms. The extension of this approach would be to model single organisms, and connect these models to one community model.

Producing a good model of a single organism is the first step in this process. There exist high-throughput methods, like ModelSEED [186], Pathway Tools [187] or KBase [188], which can automatically construct a genome scale metabolic model (GSMM) from the given genomic information. Although such reconstructions can be of high quality, it is still likely that the model will contain errors or gaps, which need to be solved by manual curation [189].

If different models for the relevant organisms can be obtained, the next challenge is combining them. If the models are based on different databases/coming from different sources, then this could result in incongruences in the final model. While this should in general be avoided, it is sometimes necessary, because high quality models of different organisms exist (e.g. *Homo sapiens* [190], *Escherichia coli* [191]), and it is not feasible to integrate this work into the high-throughput frameworks. For such cases, an integration of different model sources needs to be performed. The challenge is to match all the metabolites that need to be shared between all relevant models. Due to different problems, like the lack of unique identifiers, matching these names is not a trivial task, can be very error prone and requires the application of specialized tools (e.g. [192]).

Different hypotheses can be tested after a multi-organism model has been finally generated (e.g. [193, 194]). One of the first approaches should be to investigate ecological compatibility. This can be done for example via reverse ecology [195], by matching the metabolites in the different organisms to each other to see possible interconnections and metabolic dependencies. More advanced challenges are to actually simulate this metabolism. Finding the target, the objective function of a model, will depend on the underlying biology. Maximization of biomass is often used in single-organism models [196] (among others) and has also been used in multi-organism models (e.g. [193, 197]). This is not applicable in all cases because for example competition or parasitic relationships can exist in an ecosystem and often the objective is not to maximize the biomass of the competitors in the surrounding. Therefore, more sophisticated methods like D-OptCom [198] have been developed, which break the community optimization problem into multiple single problems. These consist of smaller optimization problems for each community member, and the main problem is to optimize the community. Others have extended this to even include spatial structures [199]. This allows the simulation of each bacterium’s growth independently, giving a more realistic result than simulating community growth.

Metabolic models are not the only models which can be employed, metabolism is also not the only type of process which can be simulated, and the bacterial level is not the only scale which can be considered. Different kinds of kinetic models of the metabolism have been developed, some especially for the gut [200, 201], and also for related ecosystems [202], but this field is still in its infancy. The mentioned models also simulate metabolism, predicting the flow of carbohydrates into acids or extracellular polysaccharides, including different non-metabolic parameters like peristaltic movement of the gut. Also non-metabolic models exist, with the focus on antibiotic resistance in the gut [203] or the succession of organisms in the gut [204]. As it can be seen, the field is still far away from a comprehensive virtual gut model. In fact, already the whole cell model [205] is extremely complex, and contains for example different scales which might be lacking full integration into the model. With all the different factors to consider, integrating more data into the models with proper feedback systems, until up to the ecosystem level, will probably be a research objective for many years to come [206].

### How to change the sum, and its parts

Modelling cannot be only done in silico. With synthetic biology, artificial model systems of the gut environment have been created [207]. These models vary in their complexity and capabilities to simulate the environment. It is important to differentiate which part of the gut is modelled, if there need to be multiple compartments, and if for example each of them needs to be pH controlled. These systems were shown to simulate parts of the gut appropriately [208], and [209] showed the contributions of intestinal movement to the development of inflammation in the gut.

But since these systems do not (yet) perfectly model the gut, final proof has often to be provided from animal models. Gnotobiotic animals [210] offer the possibility for controlled interventions. In contrast to the in vitro systems, the in vivo system will be able to incorporate all the necessary factors to evaluate gut functioning. Inoculation of the sterile animals with a defined microbiota (“synthetic ecology”) allows studying the niches of specific bacteria [211, 212], the development of the microbiota over time [204], during development [213] and the interactions between different bacteria [58, 60, 214]. Gnotobiotic animal models have also been used, as mentioned earlier, to show that the microbiota does not only change with obesity, but that it also contributes to it [33, 37, 38, 215].

At the end, it still needs to be taken into account that animal models do not represent humans, and ways to influence our gut microbiota in a rational way are only partially understood. One of these rational methods is the gastric bypass. It is one of the last resorts for morbidly obese patients to lose weight, will have a significant effect on a subject carbohydrate consumption and will alter the gut microbiota in different ways [216–219] (mainly an increase in Gammaproteobacteria), due to different changing factors like for example the distribution of bile acids. This is the most drastic method for a targeted microbiota change besides antibiotics and faecal transplantation. The latter has been used to treat severe diseases like *Clostridium difficile* infection (e.g. [220, 221]) or Ulcerative Colitis [222]. Faecal transplantation replaces a patient’s gut microbiome with that of healthy donors, however, mechanisms underlying success or failure of the treatment have not yet been fully understood in all cases. The main factors do not only include the gut microbiota itself or the host genetics [174], but potentially also other factors like excreted metabolites [223, 224]. Due to the difficulties of understanding the mechanisms, it has not yet been possible to rationally design a medicine from this therapy, which would simplify the production and legal issues [225, 226], but progress is likely to be made within the coming years [184, 227].

Microbiome changes do not only have clinical impact. Pre-clinical applications are also possible. Nutritional methods can be rationally employed, without having dramatic impact on the everyday life and include mainly pre- and probiotics. The substances and microorganisms consumed are not new, and have been already consumed for millennia, e.g. as fermented milk products. But also their mode of action is not fully understood, and in some cases their usefulness is even debated [228]. Probiotics like *Lactobacillus* and *Bifidobacterium* (e.g. [229, 230]) might act in different ways. Tested hypotheses are that they might change the gut environment to make it inhospitable for pathogens [231, 232], produce antimicrobial compounds like SCFAs [233–235], alter the composition by releasing compounds from otherwise indigestible substrates (e.g. prebiotics) [229, 236] or reverse/prevent dietary effects [237, 238]. But even in such controlled setups it is too simple to attribute changes to single organisms, since the breakdown of prebiotics (leading to “postbiotics”, which might be the actual bioactive compound) can involve multiple organisms (see for example the summary about quercetin in [239]).

## Conclusions

The currently available body of research has shown that it is important to take the ecosystem as a whole into account to understand its health implications. Recently, this trend is increasingly being picked up. After the first human genomes were sequenced, it was believed that it would change how medicine works. It was thought that every aspect of a human would be understood and that all treatments would be personalized [240, 241]. Although personal genome sequencing is still on the rise [242], this prediction has not turned out to be fully true [243], although it should be noted that there have also been significant successes (see for example table 1 in [244]). While we for sure do not yet fully understand the human genome [245], we need to be aware now that it is not the only factor. The personal well-being is not only influenced by our genetic traits. Our complete ecosystem, the whole holobiont, needs to be taken into account. It is already clear that we cannot understand obesity if we do not understand our microbiome, and if we do not understand its connections to the host. With discoveries like the enterotypes [165] (caution for the results [168], as they have been discussed widely, with the notion that gradients are more likely than separate clusters), the next step after the personal genome might even be the personalized metagenome (and the first companies are even trying to market it). If people have different microbiomes, they might need to be treated differently to combat for example obesity. With enough data, and the understanding of its meaning, it might also be possible to prevent this lifestyle epidemic, in combination with personalized nutrition, as it is even already becoming potentially feasible [175]. We might also be able to go further, and even prevent diseases. The preventive measures are normally not part of the regular mainstream medicine, but ideas exist how incorporate preventive measures, pioneered as “4P medicine” (predictive, preventive, personalized, participatory) [246, 247]. If we know a person’s microbiome, we will be able to predict if they are for example more prone to obesity or other risk factors (which is for some disease states already possible [160, 161]). If we understand the functionality, we will be able to take countermeasures with dietary interventions like pre- and probiotics. Since all these ecosystems are different, this approach will need to be personalized. Not only to take the personal genome and the personal microbiome into account, but also the compatibility with lifestyle, because even the best treatment might not suffice if a subject consumes by default a high fat “western diet” without any exercise. And this is all not possible, if the population does not participate. This approach will rely on everyone’s personal data, which needs to be acquired. And it will only work, if the results are communicated clearly.

All of these points are future challenges. We do not yet fully understand the microbiome. With diet we are taking counter measures, but not always in rational ways. Medicine is already personalized, but not all treatments have the necessary data to be personalized. And while communication can already work (e.g. the whole “quantified self” movement is relying on achievements being communicated back), it is not always the case, and wrong communication, resulting in wrong expectations, will even discourage the users (e.g. [248]). The researchers in the microbiome field need to be aware that this hype can also happen to the microbiome [249, 250].

Current microbiome research aims to overcome some of these challenges. Obesity research is likely to contribute in the close future to a better understanding of the underlying mechanisms, and the 4P medicine might partially become achievable in not too distant future, leading to better health and combating epidemics like obesity.

## Additional file

Additional file 1: Supplementary Materials and Methods. Description on how data for Figs. 1 and 2 were obtained. (DOCX 16 kb)
