# Supplementary material for: Studying microbial functionality within the gut ecosystem by systems biology
Source: Genes Nutr. 2018 Mar 6;13:5. doi: 10.1186/s12263-018-0594-6 (PMC5840735; doi:10.1186/s12263-018-0594-6)
Supplement: Supplementary file 1 — Supplementary Materials and Methods. Description on how data for Figs. 1 and 2 were obtained. (DOCX 16 kb) [file 12263_2018_594_MOESM1_ESM.docx]

**Appendix Material & Methods**

Data was extracted by querying Pubmed (11.01.2017) and downloading results as csv files.

All lines containing “review” were excluded.

To gather data on gut related meta-omics publications, Pubmed was queried for different terms. To get approximate data for any type of meta-omics and gut related topics, the following was searched for.

Any of these terms: Metagenomics, metagenomic, metagenome, metatranscriptomics, metatranscriptome, metatranscriptomic, metaproteome, metaproteomics, metaproteomic, meta-metabolome, metametabolomics

Together with any of the following terms: Gut, GIT, intestine, intestinal, feces, faeces, fecal, faecal, stool

Queries were then further restricted to distinguish between test subjects, to be specific:

1. Humans
2. Pig, pigs, swine, piglet, piglets
3. Mouse, mice, rat, rats, rodent
4. Zebrafish, zebrafishes, fish, fishes
5. Termite, termites, insect, insects, drosophila

To distinguish between gut sites, the queries were restricted to:

1. Duodenum
2. Jejunum
3. Ileum
4. Caecum
5. Appendix
6. Colon
7. feces, faeces, fecal, faecal, stool

Treatments were distinguished by the following terms:

1. prebiotics, prebiotica, prebiotic
2. probiotics, probiotica, probiotic
3. fecal transplant, faecal transplant, fecal transplantation, faecal transplantation, donor feces, donor faeces, faecal infusion, fecal infusion
4. gastric bypass, gastric surgery, RYGB, post-gastric-bypass, bariatric surgery

Furthermore Pubmed was queried for all articles containing the words clone, clones, fosmid, microarray, microarrays, and these articles were excluded.

To summarize articles per publisher, the following actions were performed.

To count all BMC journals, all journals starting with “BMC” were summed up, together with the journals Microbiome, Genome Biol, Biotechnol Biofuels and Stand Genomic Sci.

To count all Nature journals, all journals starting with “Nat “ were summed up, as well as Nature, ISME J and Sci Rep.

For the sum of all PLoS journals, all journals starting with PLoS were counted.
